# Mechanical Modelling of the Plastic Flow Machining Process

**DOI:** 10.3390/ma11071218

**Published:** 2018-07-16

**Authors:** Viet Q. Vu, Yan Beygelzimer, Roman Kulagin, Laszlo S. Toth

**Affiliations:** 1 Université de Lorraine, CNRS, Arts et Métiers ParisTech, LEM3, F-57073 Metz, France; 2 Laboratory of Excellence on Design of Alloy Metals for low-mAss Structures (DAMAS), Université de Lorraine, F-57073 Metz, France; vqviet@tnut.edu.vn; 3 Donetsk Institute for Physics and Engineering Named after O.O. Galkin, National Academy of Sciences of Ukraine, 03680 Kyiv, Ukraine; yanbeygel@gmail.com; 4 Institute of Nanotechnology (INT), Karlsruhe Institute of Technology (KIT), 76021 Karlsruhe, Germany; kulagin_roma@mail.ru

**Keywords:** lateral extrusion ratio, Finite Element (FE) simulation, analytical modelling, plastic flow machining, back pressure

## Abstract

A new severe plastic deformation process, plastic flow machining (PFM), was introduced recently to produce sheet materials with ultrafine and gradient structures from bulk samples in one single deformation step. During the PFM process, a part of a rectangular sample is transformed into a thin sheet or fin under high hydrostatic pressure. The obtained fin is heavily deformed and presents a strain gradient across its thickness. The present paper aims to provide better understanding about this new process via analytical modelling accompanied by finite element simulations. PFM experiments were carried out on square commercially pure aluminum (CP Al) billets. Under pressing, the material flowed from the horizontal channel into a narrow 90° oriented lateral channel to form a fin sheet product, and the remaining part of the sample continued to move along the horizontal channel. At the opposite end of the bulk sample, a back-pressure was applied to increase the hydrostatic pressure in the material. The experiments were set at different width sizes of the lateral channel under two conditions; with or without applying back-pressure. A factor called the lateral extrusion ratio was defined as the ratio between the volume of the produced fin and the incoming volume. This ratio characterizes the efficiency of the PFM process. The experimental results showed that this ratio was greater when back-pressure was applied and further, it increased with the rise of the lateral channel width size. Finite element simulations were conducted in the same boundary conditions as the experiments using DEFORM-2D/3D software, V11.0. Two analytical models were also established. The first one used the variational principle to predict the lateral extrusion ratio belonging to the minimum total plastic power. The second one employed an upper-bound approach on a kinematically admissible velocity field to describe the deformation gradient in the fin. The numerical simulations and the analytical modelling successfully predicted the experimental tendencies, including the deformation gradient across the fin thickness.

## 1. Introduction

Ultrafine-grained (UFG) material structure exhibits significantly high strength together with satisfactory ductility, and other attractive service properties such as superplasticity, good fatigue strength, and high wear resistance, among others [1]. The possibility of producing UFG materials has been successfully established for various severe plastic deformation (SPD) techniques. SPD processes are defined as metal forming processes that impose a very large plastic strain on a processed sample to produce UFG materials under very high hydrostatic pressure. Some examples of SPD processes are high pressure torsion (HPT), equal-channel angular pressing (ECAP), multi-axial forging, and twist extrusion, among others [2]. The use of gigantic hydrostatic pressure permits to reach extremely large strains because it reduces the tendencies to form cracks [3].

Concerning sheet materials with UFG structure, several SPD techniques have been proposed. They are the following: accumulated roll bonding [4], asymmetric rolling [5], continuous confined strip shearing [6], equal channel angular sheet extrusion [7], con-shearing process (CSP) [8], repetitive corrugation and strengthening [9], and equal channel angular rolling [10]. However, all these techniques require a sheet form as the initial sample and only transform their microstructure. The production of UFG sheet materials directly from bulk coarse-grained blanks will expand the range of technologies and open new possibilities. Thus, methods that allow for this are of considerable interest. Traditional metal forming approaches, such as rolling or direct extrusion, require considerable energy and very powerful machines for forming UFG sheet materials from bulk billets by cold plastic deformation. Processes where the applied power is reduced are of high interest.

A recently developed process, named large strain extrusion machining (LSEM, [11,12]), is a process developed in this technological direction. It is a modified machining process in which continuous metal chips are produced with UFG structure because of the large imposed strain. In this process, the chip formation is controlled by a constraining channel that is placed on the chip. Yet another new process, called plastic flow machining (PFM), for producing UFG sheet materials, was introduced by the present authors in the literature [13,14]. This process is able to produce sheet materials with ultrafine and gradient structures from bulk samples in a single extrusion step. During the PFM process, a surface layer of a bulk sample is severely deformed under a large hydraulic pressure to transform it into a thin sheet or fin with large imposed shear strain with strain gradient across its thickness. The advantages of PFM are manifolds. First, thanks to the large hydrostatic stress in the deformation zone; shear bands, segmentation, micro-cracks, and voids are suppressed during the fin formation. Second, a UFG structure and simple shear textures are obtained in the PFM-produced fin, providing a significant increase in strength and formability. For example, commercial pure aluminum (CP Al) fins produced by PFM have tensile strength increased by a factor of three and produce a Lankford coefficient of 0.92, which is greater than that in conventional rolling (where it varies between 0.5 and 0.85 [14]). Obtaining a high value of this coefficient is very important for improving the formability of aluminum sheets.

In this paper, we present the results of a further study on the PFM process. Numerical simulations and new analytical modelling are presented and their outcomes are compared with experimental results. The objective of this study is to provide a deeper understanding about this new process, such as the dependence of the fin formation efficiency on the die geometry and the reasons for the formation of a gradient structure within the fin.

## 2. Fundamental Principles of PFM and Experiments

**Figure 1:**
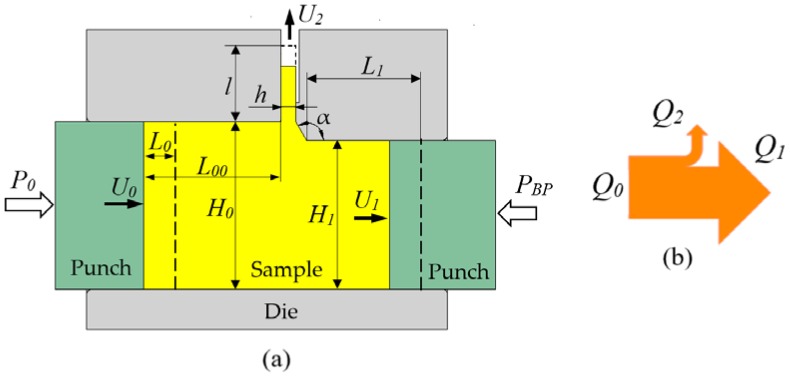
Fundamental principles of the plastic flow machining (PFM) process. (**a**) Die geometry; (**b**) Metal flow.

The fundamental principles of the PFM operation are given in the previous work [14]; they are illustrated here in Figure 1. The initial square billet sample with height $H_{0}$ is fit into the horizontal square channel die. In the middle part of the horizontal channel, the height of the channel is reduced slightly from $H_{0}$ to $H_{1}$. Right at the reduction point, a lateral channel with width size *h* is opened. The die edge connecting the horizontal and lateral channels is inclined at an angle *α* with respect to the horizontal channel. A small step is made along the lateral channel to reduce friction of the outgoing fin with the channel’s surface. The sample is pressed on its left side by a pressing punch, which moves at a constant velocity $U_{0}$. On the right side of the sample, a constant back-pressure $P_{BP}$ is applied by another punch. Because of the geometry of the die, there are two extrusion processes: lateral and forward extrusions. The lateral extrusion produces the thin sheet or fin, and the forward extrusion moves the material horizontally.

One of the key features of a lateral extrusion process—pointed out already by Hill (see Figure 33 in the literature [15])—is that a high-pressure zone is generated at the intersection zone between the lateral and horizontal channels, provided that the thickness reduction $r=(H_{0}-H_{1})/H_{0}$ is sufficiently small (between about 0.01 and 0.15). This high pressure drives the material into the lateral channel and produces heavy deformation. During processing, the incoming flow $Q_{0}$ is separated into two flows: $Q_{0}=Q_{1}+Q_{2}$. These flows can be defined as the volumes passing the cross sections per unit time: 

Pressing flow:

(1) $$Q_{0}=U_{0}.D.H_{0}$$

Forward extrusion flow:

(2) $$Q_{1}(t)=U_{1}(t).D.H_{1}(t)$$

Lateral extrusion flow:

(3) $$Q_{2}(t)=U_{2}(t).D.h(t)\;$$

Here, $U_{0}$, $U_{1}(t)$, and $U_{2}(t)$ are the respective material flow velocities and *D* is the transverse dimension of the workpiece. Note that the $Q_{1}$ and $Q_{2}$ material flows are not necessarily constant during the whole extrusion process, so we consider them as a function of time. Indeed, while $Q_{0}$ is imposed to be constant by the pressing punch, which moves with a constant speed, there is no such condition for the other two flows, $Q_{1}$ and $Q_{2}$. In order to define the relative amount of material moving into the lateral channel, a parameter called lateral extrusion ratio x is introduced:

(4) $$x(t)=Q_{2}(t)/Q_{0}$$

Therefore,

(5) $$Q_{1}(t)=\left(1-x(t)\right)Q_{0},\;Q_{2}(t)=x(t)Q_{0}$$

Combining Equations (1), (3), and (4), we obtain the following:

(6) $$x(t)=\frac{U_{2}(t).h}{U_{0}H_{0}}$$

The $U_{0}$ and $U_{2}(t)$ velocities are defined as follows:

(7) $$U_{0}=dL_{0}(t)/dt,\;U_{2}(t)=dl(t)/dt$$

Which results in the following:

(8) $$x(t)=\frac{l(t).h}{L_{0}(t).H_{0}}$$

The maximum possible value of *x* is 1, which occurs when the back-pressure punch is fixed. In this case, PFM transforms into a kind of non-equal channel angular pressing process (NECAP [16,17]).

It is important to investigate the dependence of the lateral extrusion ratio on the die geometry and back-pressure, because this ratio determines the efficiency of the fin formation. A larger value of this ratio means a larger amount of material flowing into the lateral channel to form a longer fin. In industrial applications, one should design a proper die geometry and back-pressure so that the lateral extrusion can be operated with high efficiency.

Several experiments were carried out on commercial pure (CP) Al-1050 at room temperature to examine the variations in the *x* parameter. As the evolution of l(t) was not possible to follow during the test, the following *x* value was defined from the experiments:

(9) $$X=\frac{l(T).h}{L_{0}(T).H_{0}}$$

where *T* is the total time of the extrusion operation.

**Figure 2:**
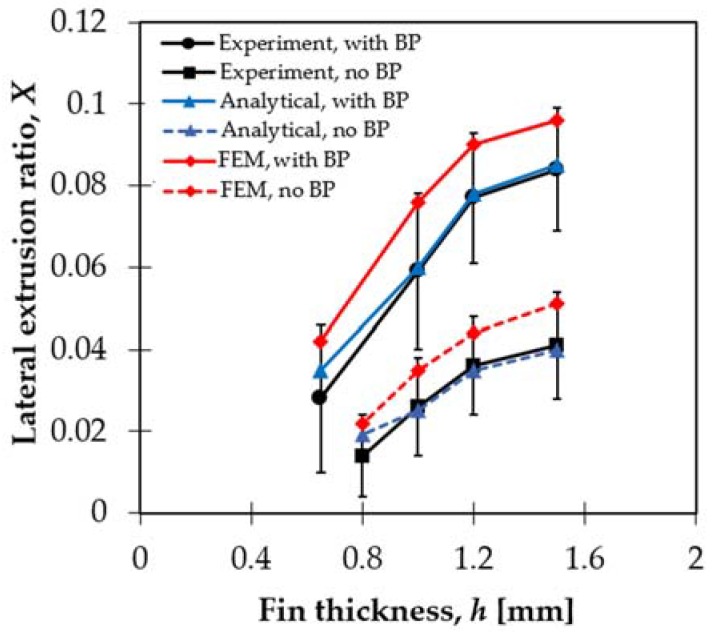
The dependence of the lateral extrusion ratio on the gap-width, for a back-pressure (BP) of 110 MPa, as well as without back-pressure. FEM—finite element modeling.

The experiments were conducted in a modified equal channel angular pressing machine equipped with two punches that were controlled by a hydraulic system. The force capacity of each hydraulic punch was 72 tons. Dies and punches of the machine were made of high-alloy tool Z160CDV12 steel subjected to heat treatment to have a yield limit about 2 GPa and a hardness of 58 HRC. The pressing punch on the left side of the sample was controlled to move at a constant speed of 1 mm/s. When back-pressure was applied, the punch on the right side was loaded at a constant pressure of 110 MPa. In order to examine the effect of back-pressure, testing was also carried out without applying back-pressure. Only the gap-width *h* was varied, between 0.2 and 1.5 mm, all other geometrical parameters of the die were kept constant; $H_{0}=20$ mm, $H_{1}=18$ mm, and die angle α=120°. The results obtained for the lateral extrusion ratio *X* for eight testing conditions are shown in Figure 2.

It can be seen in Figure 2 that the lateral extrusion ratio increased with the increase of the gap-width in both cases, with and without back-pressure, and *x* was larger with back-pressure. The fin was not formed for *h* less than 0.8 mm without BP and 0.6 mm with BP, so only direct extrusion took place for small *h* values. In these cases, a dead-metal-zone adjacent to the lateral channel entry was formed, which hindered the material flow into the lateral channel. The results in Figure 2 clearly show that the fin formation was improved by conducting PFM under back-pressure and that the gap-width size had to be above a minimum value.

As in the modeling below, we will obtain *x* as a function of time (or $L_{1}$), *X* can be derived as follows from the modeling. The total length of the fin is as follows:

(10) $$l(T)=\int_{0}^{T}U_{2}(t)dt$$

Now, using the following relations:

(11) $$U_{2}(t)=x(t)\frac{H_{0}U_{0}}{h},\;dt\frac{dL_{1}(t)}{U_{1}(t)}=\frac{H_{1}dL_{1}(t)}{\left(1-x(t)\right)H_{0}U_{0}}$$

We obtain the following:

(12) $$l(T)=\frac{H_{1}}{h}\int_{0}^{L_{1}}\frac{x(L_{1})}{1-x(L_{1})}dL_{1}$$

Replacing this relation into Equation (9), we obtain the following:

(13) $$X=\frac{H_{1}}{L_{0}(T).H_{0}}\int_{0}^{L_{1}}\frac{x(L_{1})}{1-x(L_{1})}dL_{1}$$

Therefore, in the modeling, we will determine $x(L_{1})$, then apply the integral (13) to derive the *X* value, which can be directly compared to the experimental *X*.

## 3. Numerical Simulation of the PFM Process

The commercial finite element code, DEFORM-2D/3D V11.0 FE, was employed to perform finite element (FE) simulations to obtain velocity fields and the distributions of stress and strain in the sample during processing. The FE simulations were performed in an incremental manner using the updated Lagrangian approach together with the Newton–Raphson iteration method. The die components were set as rigid bodies. The sample was constructed by 12,000 four-node quadrilateral deformable elements. Adaptive meshing was employed for accommodating large strains in the deformation zone near the entry of the lateral channel. The boundary conditions were set similar to the experiments. The pressing punch velocity was set at a constant value of 1 mm/s. The back pressure was set at two different values: 30 MPa and 110 MPa. The material behavior was taken von Mises isotropic type using Hollomon’s power law [18]: $\sigma =180\varepsilon_{vM}^{0.23}\mathrm{MPa}$, where σ and $\varepsilon_{vM}^{}$ are the von Mises equivalent stress and equivalent strain, respectively. The hardening parameters were identified using the experimental stress-strain curve obtained for Al1050 in the literature [19]. The frictional shear stress τ was modeled by the Siebel friction law [18]: τ=μσ, where σ is the flow stress, employing a friction coefficient of μ=0.2.

Regarding possible thermal effects, in general, during SPD processes at room temperature, there is no significant increase in temperature. The maximum observed temperature increase was about 5 °C for aluminum. For example, Alexander and Langdon [20] pointed out that the temperature increase during HPT, which is the most severe SPD technique, was only about 5 °C in aluminum. The main reason for this is that the processing velocities are not high in SPD; in the present study, the pressing punch moved only 1 mm/s. Therefore, thermal effects are small and can be neglected in the modeling.

**Figure 3:**
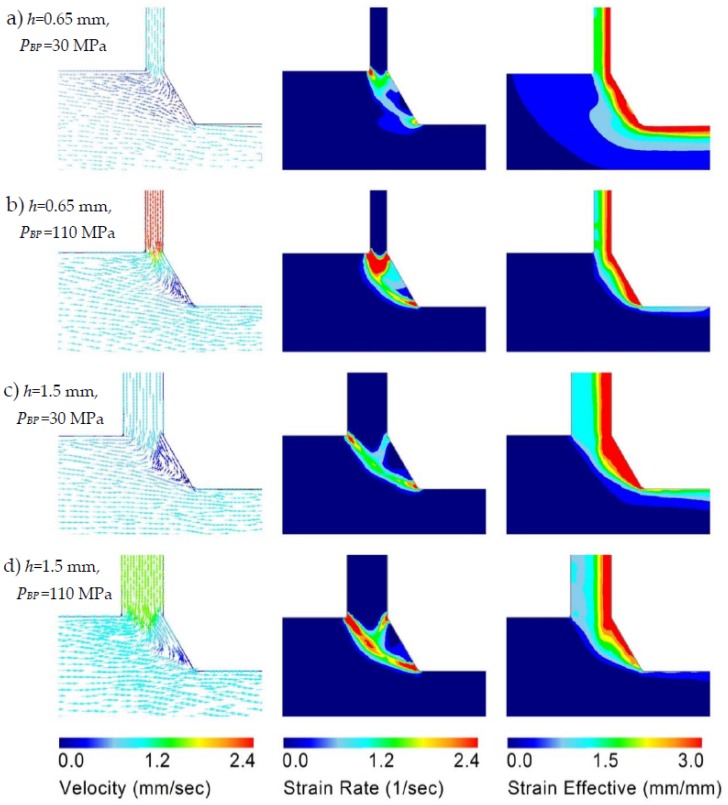
Results of finite element simulations of the PFM process for the parameters: *H*_0_ = 20 mm; *H*_1_ = 18 mm; *α* = 120°. The magnitudes of the velocity, strain rate, and effective strain (equivalent strain) are indicated by the respective color codes (the maximum value of the equivalent strain is about 4 in all cases).

Figure 3 presents the results of the FE simulations obtained for the velocity field, the strain rate, and the accumulative strain distribution for two back pressure values (30 MPa and 110 MPa) and for two gap-width sizes (*h* = 0.65 mm and 1.5 mm). The velocity fields for both gap-widths show that an increase in back pressure leads to an increase in the material velocity within the lateral channel. Therefore, the fin is longer with back-pressure. This is in agreement with the experimental results in Figure 2.

For each case in Figure 3, there is a low velocity zone near the die edge, where the velocity is very small, but non-zero (the blue color code indicates the low velocity). One can consider such zones as “dead metal zones” (DMZ), which are commonly seen in extrusion processes because of the friction between the metal and the die walls. If the gap-width for the lateral channel is too small, then the DMZ can be large enough to prevent the occurrence of the lateral material flow.

The effective strain distributions in the fin in Figure 3 show deformation gradients across the fin thickness. The reasons for this strain gradient will be revealed by an analytical model in the next section. The strain rate distributions in Figure 3 show the zones where high deformation takes place; the obtained features are useful to construct the analytical model.

## 4. Analytical Modeling of the PFM Process

For a better understanding of the PFM process, two analytical models were established. In these models, the material is considered rigid-plastic, for simplicity. Indeed, our CP Al material was already in a hardened state, so only small further strain hardening took place during PFM. As the sample is constrained by the die walls in the transverse direction (perpendicular to the plane of Figure 1), there is no strain along this direction and this permits us to adopt a plane strain state. In the first model, we aim to model the dependence of the lateral extrusion ratio *x* on the back-pressure and the width of the slit; we used the “minimum properties of an actual velocity field” principle (p. 332 in the literature [21]). The actual value of *x* must minimize the total rate of work as we employed the variational principle of mechanics. For the second model, where we study the deformation gradient within the fin, we used the upper-bound approach applied on a kinematically admissible velocity field, formed by rigid blocks [22].

### 4.1. Model for the Lateral Extrusion Ratio

In this section, a model is presented for predicting the lateral extrusion ratio as a function of the back-pressure and the gap-width.

With the help of the upper bound theorem [21], the *x* value, which expresses the fraction of the material of the workpiece that flows to form the fin in a time increment, can be determined by minimizing the total power generated during the PFM process, it is given by the following:

(14) $$W=W_{1}+W_{2}+W_{BP}+W_{fr}$$

where $W_{1}$ and $W_{2}$ are the plastic powers for the forward and lateral extrusions, respectively; $W_{fr}$ is the friction power caused by the contact between the sample and the die; and $W_{BP}$ is the power produced by the back pressure.

(15) $$W_{BP}=P_{BP}H_{1}DU_{1}$$

Assuming that the whole sample volume is in plastic state during the extrusion process, $W_{1}$ and $W_{2}$ are defined as follows:

(16) $$W_{1}=a_{1}Q_{1},\;W_{2}=a_{2}Q_{2}$$

Here, $a_{1}$ and $a_{2}$ are the plastic work per unit volume, which, for isotropic material, can be obtained from the flow stress $\sigma_{0}$ and the equivalent plastic strain $e_{eq}$ as follows [21]:

(17) $$a=\sigma_{0}e_{eq}$$

According to Equations (4) and (5), the PFM process can be represented as a lateral extrusion of the workpiece, where the surface layer with thickness $xH_{0}$ flows into the lateral channel, and a simultaneous forward extrusion of the workpiece with a thickness $\left(1-x\right)H_{0}$. The width of the lateral channel is *h*, and the direct channel’s thickness is $H_{1}$. If $\left(1-x\right)H_{0}>H_{1}$, an elongation of the specimen takes place in the direct extrusion channel, and the equivalent plastic strain $e_{eq1}$ can be determined by the plain strain extrusion formula, adapted from the literature [22].

(18) $$e_{eq1}=\frac{2}{\sqrt{3}}\ln\frac{\left(1-x\right)H_{0}}{H_{1}}$$

Employing the above-defined *r* parameter to simplify the expression, we obtain the following:

(19) $$e_{eq1}=\frac{2}{\sqrt{3}}\ln\frac{1-x}{1-r}$$

Note that there is no direct extrusion strain if x=r. This happens when all material contained in the upper part of the workpiece (the $H_{0}-H_{1}$ thickness layer) goes into the fin. When x>r, then the material in the *Q*_1_ flow is under compression strain. That requires a very high back-pressure. The most probable case is, however, when x<r, for which case the direct extruded material is under tensile strain. In order to keep the strain constantly positive for the plastic work calculation, we use the absolute value in Equation (19):

(20) $$e_{eq1}=\frac{2}{\sqrt{3}}\left|\ln\frac{1-x}{1-r}\right|$$

Plugging this relation into Equation (12), one obtains the following:

(21) $$a_{1}=\frac{2\sigma_{0}}{\sqrt{3}}\left|\ln\frac{1-x}{1-r}\right|$$

Taking into account this relation, we obtain from Equation (16) the following formula for the plastic power of the forward extrusion:

(22) $$W_{1}=\frac{2\sigma_{0}}{\sqrt{3}}Q_{1}\left|\ln\frac{1-x}{1-r}\right|$$

**Figure 4:**
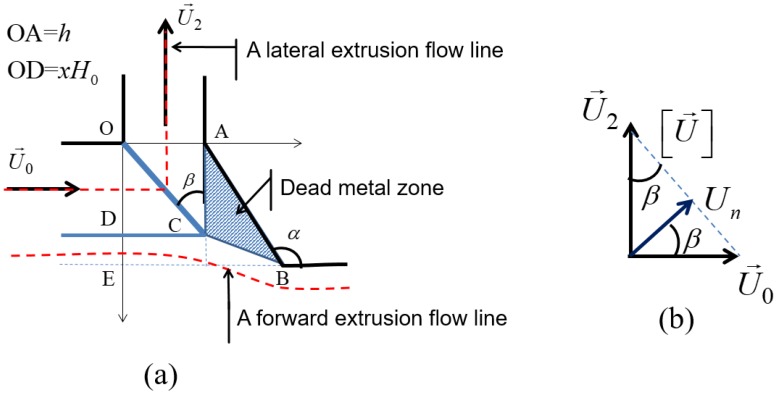
Schematic of a kinematically admissible velocity field for the flow into the lateral channel showing the dead metal zone (**a**); the velocity hodograph is shown in (**b**).

To estimate the average equivalent strain for the lateral extrusion, we simplify the complex material flow obtained from the FE simulations by a simple non-equal channel angular pressing flow process [16,17], by defining a dead metal zone as indicated in Figure 4. The entry dimension is the OD distance, and the exit dimension is OA = *h*. As mentioned before, during lateral extrusion, the material flows from the surface layer of the bulk sample into the lateral channel have the thickness $xH_{0}$, this leads to $OD=xH_{0}$. In this simplified kinematically admissible velocity field, there is only one velocity discontinuity line, the OC segment. 

When crossing the velocity discontinuity segment, the material acquires an equivalent plastic strain equal to the following (see [22]):

(23) $$e_{eq}=\frac{1}{\sqrt{3}}\left|\frac{\left[\vec{U}\right]}{U_{n}}\right|$$

where $U_{n}$ is the velocity component normal to the OC segment, and $\left[\vec{U}\right]$ is the velocity discontinuity vector. With the help of Figure 4b, one can write the following:

(24) $$\left[\vec{U}\right]=U_{n}\tan\beta +\frac{U_{n}}{\tan\beta }$$

Because, in the considered kinematic field, all material flows into the fin within the upper zone defined by the OD segment, the ratio *OD/H*_0_ is equal to the *x* parameter. Therefore, it follows from the geometry in Figure 4a that,

(25) $$\tan\beta =\frac{OA}{AC}=\frac{OA}{OD}=\frac{h}{xH_{0}}=\frac{\bar{h}}{x}$$

where $\bar{h}=h/H_{0}$. Finally, from Equations (23)–(25), we get the following:

(26) $$e_{eq2}=\frac{1}{\sqrt{3}}\left(\frac{x}{\bar{h}}+\frac{\bar{h}}{x}\right)$$

Note that this formula is the same as the one developed for the non-equal channel angular extrusion process for a 90° die [16], where the incoming channel thickness is $xH_{0}$ (the OD segment in Figure 4) and the outgoing one is *h*. Plugging this relation into Equation (17) one can get the following:

(27) $$a_{2}=\frac{\sigma_{0}}{\sqrt{3}}\left(\frac{x}{\bar{h}}+\frac{\bar{h}}{x}\right)$$

Taking into account this relation, from relation (16), we obtain the following formula for the plastic power for the lateral extrusion:

(28) $$W_{2}=\frac{\sigma_{0}}{\sqrt{3}}Q_{2}\left(\frac{x}{\bar{h}}+\frac{\bar{h}}{x}\right)$$

With the help of Equations (15), (22), and (28), Equation (14) can be developed as follows:

(29) $$W=\frac{2\sigma_{0}}{\sqrt{3}}Q_{1}\left|\ln\frac{1-x}{1-r}\right|+\frac{\sigma_{0}}{\sqrt{3}}Q_{2}\left(\frac{x}{\bar{h}}+\frac{\bar{h}}{x}\right)+P_{BP}H_{1}DU_{1}+W_{fr}$$

It is useful to obtain dimensionless quantities, so for this purpose, we divide both sides of this equation by $\sigma_{0}DH_{0}U_{0}$:

(30) $$\frac{W}{\sigma_{0}DH_{0}U_{0}}=\frac{2\sigma_{0}}{\sqrt{3}}\frac{Q_{1}}{\sigma_{0}DH_{0}U_{0}}\left|\ln\frac{1-x}{1-r}\right|+\frac{\sigma_{0}}{\sqrt{3}}\frac{Q_{2}}{\sigma_{0}DH_{0}U_{0}}\left(\frac{x}{\bar{h}}+\frac{\bar{h}}{x}\right)+\frac{P_{BP}H_{1}U_{1}}{\sigma_{0}H_{0}U_{0}}+\frac{W_{fr}}{\sigma_{0}DH_{0}U_{0}}$$

Taking into account Equations (1), (2), (4), and (5), we obtain the following:

(31) $$\frac{W}{\sigma_{0}DH_{0}U_{0}}=\frac{2}{\sqrt{3}}(1-x)\left|\ln\frac{1-x}{1-r}\right|+\frac{x}{\sqrt{3}}\left(\frac{x}{\bar{h}}+\frac{\bar{h}}{x}\right)+{\bar{P}}_{BP}(1-x)+\frac{W_{fr}}{\sigma_{0}DH_{0}U_{0}}\;$$

where ${\bar{P}}_{BP}=\frac{P_{BP}}{\sigma_{0}}$.

According to the principle of the minimum plastic power of an actual velocity field [21], the value of *x* is provided by a minimum of the right-hand side of Equation (31). We consider the case where x<<1 and r<<1. For this case, one can apply a first order approximation in Equation (31):

(32) $$\frac{W}{\sigma_{0}DH_{0}U_{0}}=\frac{2}{\sqrt{3}}\left(1-x\right)\left|r-x\right|+\frac{x}{\sqrt{3}}\left(\frac{x}{\bar{h}}+\frac{\bar{h}}{x}\right)+{\bar{P}}_{BP}\left(1-x\right)+\frac{W_{fr}}{\sigma_{0}DH_{0}U_{0}}$$

Now, we determine the friction-power. $W_{fr}$ can be divided into three parts:

(33) $$W_{fr}=W_{fDMZ}+W_{f0}+W_{fBP}$$

where $W_{frDMZ}$ is generated by the friction produced by the contact between the flowing material and the edge *AC* on the dead metal zone (Figure 4):

(34) $$W_{fDMZ}=\mu_{DMZ}.P_{fin}.AC.D.U_{2}$$

Combining Equations (3), (4), and (34) using $AC=OD=xH_{0}$, we obtain the following:

(35) $$W_{fDMZ}=\mu_{DMZ}P_{fin}xH_{0}D\frac{Q_{2}}{hD}=\mu_{DMZ}P_{fin}xH_{0}\frac{xQ_{0}}{h}=\mu_{DMZ}P_{fin}\frac{x^{2}{}_{0}}{\bar{h}}DH_{0}U_{0}$$

$W_{f0}$ and $W_{fBP}$ in Equation (33) are the friction powers for the left and right parts of the samples with respect to the fin’s position. These powers were calculated by employing the method of slices (see Appendix A for details):

(36) $$W_{f0}=U_{0}H_{0}DP_{0}\left[1-\exp\left(\frac{-2\mu L_{0}\left(H_{0}+D\right)}{H_{0}D}\right)\right]\;$$

(37) $$W_{fBP}=U_{1}H_{1}DP_{BP}\left[\exp\left(\frac{2\mu L_{1}\left(H_{1}+D\right)}{H_{1}D}\right)-1\right]\;$$

where *μ* is the friction coefficient for sample-die contact surfaces. This is not unknown, however, because it can be obtained from the equality of the pressures at the position of the fin, calculated from the pressure equations from the left and the right sides within the horizontal extrusion channel. The analytic expression of this friction parameter is as follows (Equation (A5) in Appendix A):

(38) $$\mu =\frac{D}{2}\ln\left(\frac{P_{0}}{P_{BP}}\right){\left[\frac{L_{1}\left(H_{1}+D\right)}{H_{1}}+\frac{L_{0}\left(H_{0}+D\right)}{H_{0}}\right]}^{-1}\;$$

Combining Equations (1), (2), (5) and (37), one can obtain the following:

(39) $$W_{fBP}=U_{0}H_{0}D(1-x)P_{BP}\left[\exp\left(\frac{2\mu L_{1}\left(H_{1}+D\right)}{H_{1}D}\right)-1\right]$$

Now, combining Equations (32), (33), (35), (36) and (39), and by considering that the friction part $W_{f0}$ in Equation (36) does not depend on x, the actual *x* value can be obtained by minimizing the function:

(40) $$f\left(x\right)=\frac{2}{\sqrt{3}}\left(1-x\right)\left|r-x\right|+\frac{x}{\sqrt{3}}\left(\frac{x}{\bar{h}}+\frac{\bar{h}}{x}\right)+{\bar{P}}_{BP}\left(1-x\right)+\mu_{DMZ}{\bar{P}}_{fin}\frac{x^{2}}{\bar{h}}+{\bar{P}}_{BP}(1-x)\left[\exp\left(\frac{2\mu L_{1}\left(H_{1}+D\right)}{H_{1}D}\right)-1\right]\;$$

where ${\bar{P}}_{fin}=P_{fin}/\sigma_{0}$.

The minimum of f(x) depends on the applied back-pressure. Three intervals of the back-pressure can be distinguished, where the solutions are as follows (see Appendix B for the mathematical details):

Mode 1:

(41) $$x=\bar{h}\left[\left(1+r\right)+A{\bar{P}}_{BP}\right]B^{-1},\;\mathrm{when}\;{\bar{P}}_{BP}<{\bar{P}}_{BP1}$$

Mode 2:

(42) $$x=r,\;\mathrm{when}\;{\bar{P}}_{BP1}\leq {\bar{P}}_{BP}\leq {\bar{P}}_{BP2}$$

Mode 3:

(43) $$x=\bar{h}\left[-\left(1+r\right)+A{\bar{P}}_{BP}\right]C^{-1},\;\mathrm{when}\;{\bar{P}}_{BP}>{\bar{P}}_{BP2}$$

Here,

(44) $${\bar{P}}_{BP1}=\left[\frac{r}{\bar{h}}B-(1+r)\right]A^{-1},\;{\bar{P}}_{BP2}=\left[\frac{r}{\bar{h}}C+(1+r)\right]A^{-1}$$

With the following:

(45) $$A=\frac{\sqrt{3}}{2}\exp\left(\frac{2\mu L_{1}\left(H_{1}+D\right)}{H_{1}D}\right);\;B=1+2\bar{h}+\sqrt{3}\mu_{DMZ}{\bar{P}}_{fin};\;C=1-2\bar{h}+\sqrt{3}\mu_{DMZ}{\bar{P}}_{fin}$$

*x* depends on the back-pressure in Modes 1 and 3, while in Mode 2, *x* is independent of the back-pressure.

**Figure 5:**
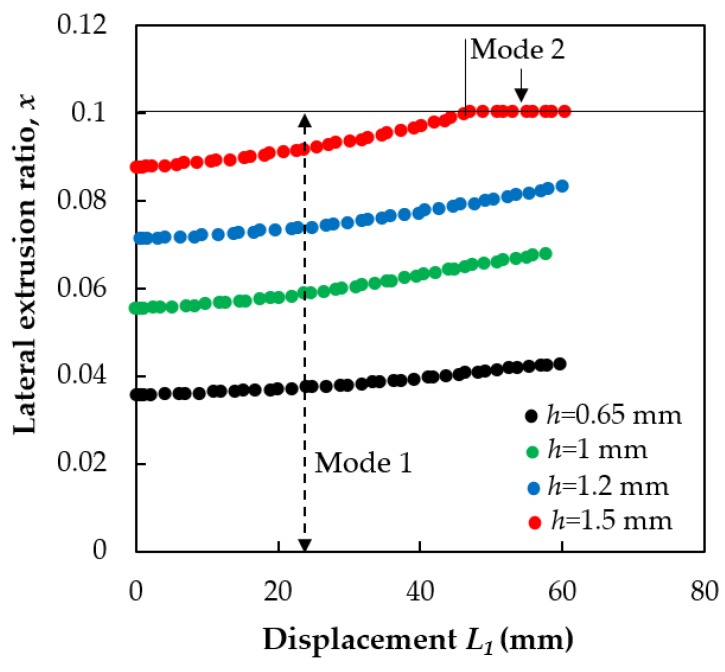
The lateral extrusion ratio *x* as the function of the displacement *L*_1_ of the back-pressure punch for four values of the gap-width *h*.

The analytical calculation was performed for the fin thicknesses of *h* = 0.65, 1.0, 1.2, and 1.5 mm by Equation (38) and Equations (41)–(45) using the measured experimental parameters together with the die geometry parameters: *H*_0_ = 20 mm and *H*_1_ = 20; flow stress: $\sigma_{0}=120$ MPa; and friction coefficient $\mu_{DMZ}=0.3$. Figure 5 shows the variation of the *x* parameter as a function of the displacement of the back-pressure punch, *L*_1_. As can be seen in the figure, *x* is generally increasing during the extrusion process. The deformation mode is generally Mode 1, except for the largest *h* value, where it reaches Mode 2.

Now, using the varying *x* parameter displayed in Figure 5 in the integral in Equation (13), we obtain the *X* value, which can be directly compared with the experiment. The results are shown in Figure 2 and Figure 8. The model reproduces the results that when back pressure is not applied, *X* decreases by about half of its value with back-pressure. The modeling results are in excellent agreement with the experiments.

### 4.2. Model for the Strain Gradient

**Figure 6:**
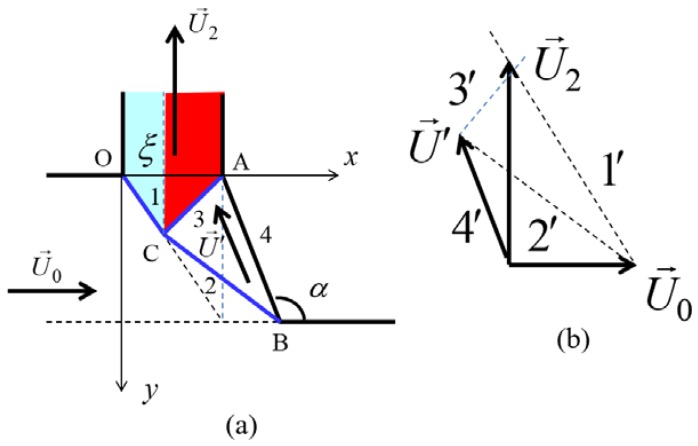
A kinematically admissible velocity field composed of three rigid blocks for the analysis of the strain distribution in the fin. (**a**) The rigid blocks with the velocity discontinuity line segments are identified by 1, 2, and 3. Left to the OCB line, the material moves with the velocity ${\vec{U}}_{0}$. The ABC triangle is moving with the velocity ${\vec{U}}^{\prime }$. Above the OCA segment, the material moves with velocity ${\vec{U}}_{2}$. α is the die angle and ξ is the abscissa of the point C. (**b**) The velocity hodograph along the CB segment.

The strain rate distribution results obtained by the FE simulations (Figure 3) suggest still another, improved kinematically admissible velocity field for an analytical estimation of the equivalent strain in PFM. We use the upper bound theory [22] to construct a kinematically admissible velocity field with rigid blocks for Mode 2 (${\bar{P}}_{BP1}\leq {\bar{P}}_{BP}\leq {\bar{P}}_{BP2}$) (Figure 6).

The dissipation power $W_{d}$ for the proposed field is given by the following formula:

(46) $$W_{d}=k\left(OC{\left|\left[U\right]\right|}_{1}+CB{\left|\left[U\right]\right|}_{2}+AC{\left|\left[U\right]\right|}_{3}\right)+mk\;ABU^{\prime }$$

where ${\left|\left[U\right]\right|}_{1}$, ${\left|\left[U\right]\right|}_{2}$, and ${\left|\left[U\right]\right|}_{3}$ are the magnitudes of the velocity discontinuity vectors at boundaries 1, 2, and 3, respectively; $U^{\prime }$ is the velocity along the *AB* boundary; and *OC*, *CB*, *AC*, and *AB* are the lengths of the corresponding segments. The last term in Equation (46) is the friction with the die-wall along the *AB* segment, where $k=\sigma_{0}/2$, and *m* is the plastic friction coefficient.

The proposed kinematically admissible velocity field has one free parameter ξ, which divides the fin into two deformation zones (see Figure 6). According to the upper bound theorem [22], the ξ value is determined by minimizing the dissipation power $W_{d}$.

The material points at the left part of the fin (Zone I) undergo shear deformation only, which is taking place at the boundary 1, while the material arriving at the right part of the fin (Zone II) undergoes shear deformations at both boundaries 2 and 3. When crossing a velocity discontinuity segment, the material acquires an equivalent plastic strain equal to the following (see [22]):

(47) $$e_{eqi}=\frac{1}{\sqrt{3}}\left|\frac{{\left[\vec{U}\right]}_{i}}{U_{ni}}\right|$$

where $U_{ni}$ is the velocity component normal to the segment *i*, and ${\left[\vec{U}\right]}_{i}$ is the velocity discontinuity vector on the segment *i* (i=1,2,3). Applying this formula to the first zone with the thickness ξ, the equivalent plastic strain is as follows:

(48) $$e_{eqI}=\frac{1}{\sqrt{3}}\left|\frac{{\left[\vec{U}\right]}_{1}}{U_{n1}}\right|.$$

In the second zone, with the thickness of (h−ξ), the von Mises strain is as follows:

(49) $$e_{eqII}=\frac{1}{\sqrt{3}}\left|\frac{{\left[\vec{U}\right]}_{2}}{U_{n2}}\right|+\frac{1}{\sqrt{3}}\left|\frac{{\left[\vec{U}\right]}_{3}}{U_{n3}}\right|.$$

The final analytical forms of Equations (47)–(49) are long to develop, yet nevertheless, quite straightforward. Here, we only present the final results that can be obtained when the formulas are fully developed and the minimization process is done.

**Figure 7:**
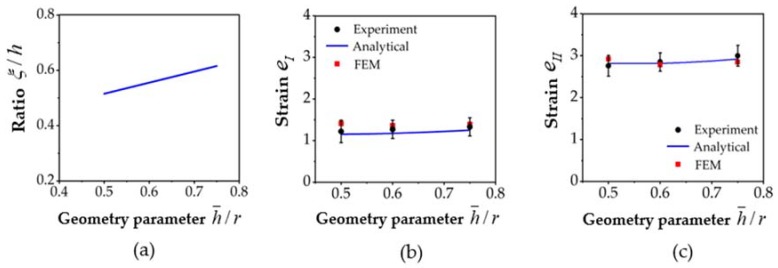
The characteristics of the strain distribution for Mode 2, obtained by the analytical model (continuous lines), and by finite element (FE) simulations (red dots) for the die angle α=120° and for the friction value of m=0.2. (**a**) The predicted width of Zone I; (**b**) the von Mises strain in Zone I; and (**c**) the von Mises strain in Zone II.

Figure 7a shows the solution for ξ/h that characterizes the partition of the two deformation zones as a function of the geometry parameter of the process $\bar{h}/r$. ξ/h varies between 0.5 and 0.62 in the range of 0.5–0.75 for the $\bar{h}/r$ parameter. Higher $\bar{h}/r$ values were not considered because they are not realistic for an experimental PFM process. The strain values in the two zones are plotted in Figure 7b,c, and as a function of the geometry parameter $\bar{h}/r$. As can be seen, the strain in Zone II is more than twice as high as that in Zone I. While the strain in Zone I increases from 1.1 to 1.3, that in Zone 2 rises from 2.8 to 3.0 when the geometry parameter $\bar{h}/r$ changes in the range of 0.5–0.75.

The strain values in Zones 1 and 2 were also determined experimentally using a master curve established for the high angle grain boundary fraction as a function of strain (see Figure 4 in the literature [23]). They are also plotted in Figure 7b,c.

Finite element simulations were also carried out to examine the strain gradient in the fin. Three cases were considered; the obtained results are displayed in Figure 7b,c. As can be seen, the results obtained by the analytical model agree with those obtained from the experiments, as well as with the FE simulations.

## 5. Discussion

In this section, the different factors that control the PFM process are analyzed using the results obtained in both the analytical and FE calculations. The three modes of the PFM process are also examined. 

### 5.1. The Lateral Extrusion Ratio

Figure 2 shows clearly that the lateral extrusion ratio *X* achieved in the case with the back-pressure assistance is about double when compared with the case without back-pressure assistance. This means that in practice, PFM should be conducted always with back-pressure so that the process is more efficient. Physically, the back-pressure applied on the right hand side of the sample (Figure 1) restricts the occurrence of the forward extrusion flow $Q_{1}$. This results in the increase of the lateral extrusion flow $Q_{2}$, because $Q_{0}=Q_{1}+Q_{2}$ is constant. Thus, it leads to a greater *X* value compared with the case without back-pressure.

**Figure 8:**
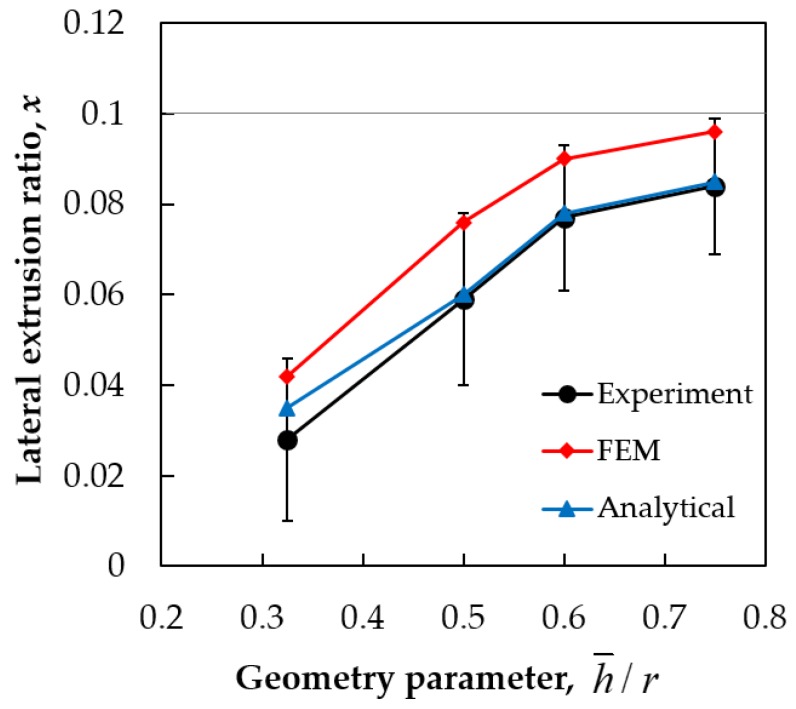
The dependence of the lateral extrusion ratio on the $\bar{h}/r$ parameter in the experiments with back-pressure of 110 MPa, by finite element simulations, as well as by the analytical model.

We now discuss the dependence of the lateral extrusion ratio on the PFM die geometry, represented by the ratio $\bar{h}/r$ in the case where back-pressure is applied (Figure 8). Note that in Figure 2, the lateral extrusion ratio is shown as the function of the gap with size *h*, which does not describe the overall geometry of the PFM die, and thus the dependence of this ratio on $\bar{h}/r$ (Figure 8) will provide a clearer view of its relationship with the die geometry. The results show that at a constant back-pressure of 110 MPa, *X* increases with the rise of the ratio $\bar{h}/r$. The results achieved from the analytical model are in excellent agreement with those received from the experiments.

There is a small difference in the values between the analytical model and the experiments in the case of small values of $\bar{h}/r$. This is because the DMZ is taken into account in a simplified way. Namely, its shape and size depend on the level of the applied back-pressure. At very small $\bar{h}/r$ values, the material flowing into the lateral channel is more restricted by the DMW. This results in a slightly smaller lateral extrusion ratio in the experiment compared with that predicted by the analytical model.

Compared with the FE simulations, the analytical model is in better agreement with the experiments. This can be attributed partly to the high strain hardening parameters used in FE simulations. In fact, the commercially pure aluminum that was used in the experiment was practically rigid-plastic, because it was already in a hardened state, so its hardening after the yield limit was very small. Therefore, the approach of the analytical model with the condition that the material is considered as rigid-plastic is appropriate and the model provides good results. In the FE simulations, treating the as rigid-plastic is difficult as it leads to non-convergences of the FE code. The other reason for the differences could be that a different friction law was used in the FE simulations (Siebel), while the analytical model used the Coulomb-law. Nevertheless, the FE results are also useful as they show that for strain hardening material, the PFM process is more efficient.

### 5.2. The Three Extrusion Modes

**Figure 9:**
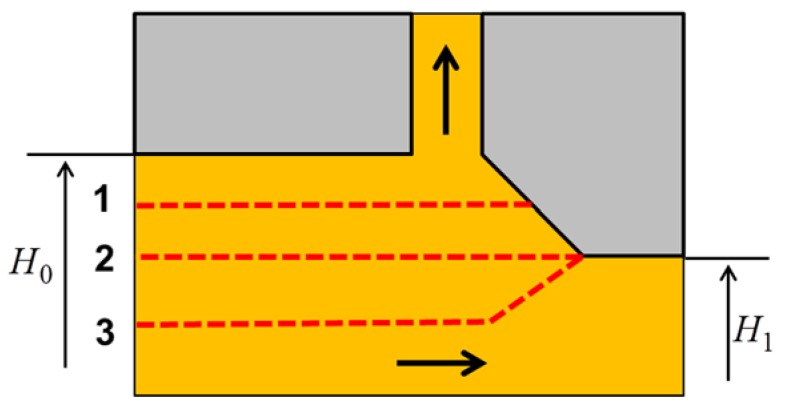
The conditional boundaries of the metal flows for the three possible PFM modes: Mode 1: ${\bar{P}}_{BP}<{\bar{P}}_{BP1}$; Mode 2: ${\bar{P}}_{BP1}\leq {\bar{P}}_{BP}\leq {\bar{P}}_{BP2}$; Mode 3: ${\bar{P}}_{BP}>{\bar{P}}_{BP2}$.

It is possible to give a geometrical interpretation of the three modes that were identified in Section 4.1 above. Simply, they differ from each other by the position of the boundary that separates the forward and lateral metal flows (Figure 9).

The separation between Modes 1 and 3 is Mode 2, for which *X = r* (Equation (42)). In this case, all material within the surface layer of the workpiece of thickness $rH_{0}$ flows into the lateral channel, so the position of the boundary line is the same as the top exit line of the workpiece; see Figure 9. This is Mode 2. In Mode 1, only a part of the top layer with thickness $\left(H_{0}-H_{1}\right)$ (above the dotted line identified by 1) goes into the lateral channel. With increasing back-pressure, the value of this part increases. If the dotted line 1 moves down to level 2, the entire $\left(H_{0}-H_{1}\right)$ layer moves into the lateral channel. When ${\bar{P}}_{BP}={\bar{P}}_{BP1}$, Mode 2 starts. In this mode, the metal flow does not depend on the back-pressure. Mode 3 starts when ${\bar{P}}_{BP}={\bar{P}}_{BP2}$. In this mode, the amount of metal flows moving into the lateral channel increases again, like in Mode 1, with an increase of back-pressure. At extremely high-back pressures, the horizontal part of dotted line 3 can go down to the bottom of the workpiece, then all the metal of the workpiece goes into the lateral channel. This extreme case corresponds to the NECAP process [16,17].

From a practical point of view, the most interesting modes of PFM are Modes 1 and 2, where the deformation is mainly concentrated in the surface layer of the workpiece and occurs by simple shear under pressure. In these modes, the power parameters of the process are small, which allows the process to be readily scaled up. The results obtained are promising for an efficient industrial application of the PFM process.

### 5.3. The Strain Gradient

The model of strain distribution (Figure 6) and the strain values calculated from this model (Figure 7) clearly show that there are two zones in the fin with different strain values in the cross-section. The obtained strain gradients correspond very well to the experimental results in this study, where the strains were estimated from the portion of next-grain large angle boundaries misorientation. There is one major difference though, which is the existence of a narrow zone (about 100 μm thick) in which the strain is very high; it is located at the right side of the fin, and not produced by the model (see in Vu et al. [14]). This zone can be attributed to the shear deformation associated with friction that is produced at the interface between the die and the workpiece. This friction also brings other effects: it enhances the strain gradient across the fin and produces a high strain zone in the top part of the bulk workpiece.

Now we examine in detail the mechanism that produces the high strain zone by friction. Because of the metal flow at the surface of the die, fresh metal comes out from the workpiece and goes into contact with the surface of the die. The sliding between the metal and the die surface can stop if the adhesion stress $\tau_{a}$ between the fresh metal and the die exceeds the shear flow stress of the metal. As a result, an intensive shear occurs at the surface layer of the workpiece. The plastic friction coefficient *m* is equal to 1 when there is no sliding. According to Prandtl [24], a surface with *m* = 1 contains an envelope of a family of slip-lines and the metal flow in close proximity to the interface is characterized by a very high strain rate (tends to infinity for ideally plastic materials). For this reason, in a narrow layer of Zone II, at the contact with the die, the metal rapidly hardens. After the shear flow stress of the metal reaches the value of $\tau_{a}$, the sliding of the metal along the wall of the die restarts. Thus, a thin, highly deformed metal layer appears near the surface of the fin. The same mechanism leads to the formation of a highly deformed thin layer on the top part of the bulk workpiece (see in Vu et al. [14]).

## 6. Conclusions

In the present work, different modeling was applied for a new SPD process, the PFM process. Two analytical models were formulated; one for obtaining the extrusion ratio, and another for the strain gradient within the produced fin. Finite element simulations were also carried out where strain hardening and friction were also taken into account. The results were compared to experimental data obtained on extrusion of commercially pure aluminum, Al 1050. From the results obtained, we can formulate the following main conclusions:

1. An analytical upper bound model was presented for modeling the lateral extrusion ratio of the PFM process. It was able to reproduce the effect of the applied back-pressure and produced results with excellent agreement with the experiment. With the help of this model, three extrusion modes were identified: Modes 1–3. The selection of the extrusion mode is determined by the applied back-pressure and one geometrical parameter, which is defined by the die geometry.
2. Another analytical model was also established to describe the strain gradient found experimentally in the fin. The model was able to produce strain values in two zones, with values near to the experiment. The third, the very high deformation zone, was interpreted with the help of the friction between the metal flow and the die wall.
3. Finite element modeling of the PFM process was carried out, where strain hardening and friction were considered. This modeling gave important information on the dead metal zone, and gave results that were generally in good agreement with the experimental observations.

## Figures and Tables

**Figure A1:**
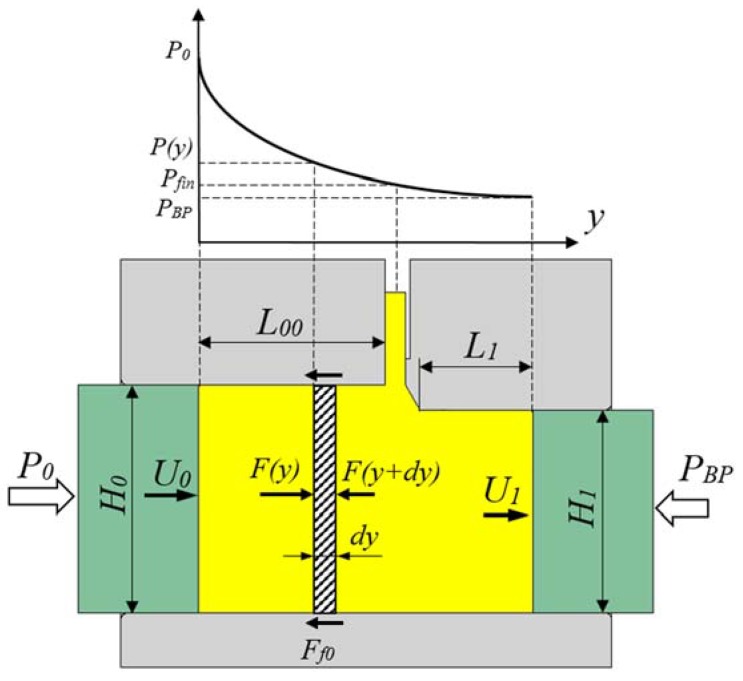
The method of slices schematically showing the stress distribution.

## Appendix A

### Power Dissipation by Friction

First, we employ the method of slices for calculating the distribution of the hydrostatic pressure within the workpiece. Then, the powers generated by the frictions will be calculated. A slice of thickness *dy* is subjected to pressure from both sides and also to the friction from the four walls of the rectangular channel (Figure A1). The equilibrium of forces provides the following:

(A1) $$F(y)-F(y+dy)-F_{f0}=0$$

where *y* is the distance from the left-surface of the sample, F(y) is the force applied by the pressure P(y), and $F_{f0}$ is the friction force applied by the die on the sample by the four walls.

Using the relations $F(y)=P(y)H_{0}D$ and $F_{f0}=2P(y)\left(H_{0}+D\right)\mu dy$, one obtains the following differential equation for the pressure distribution:

(A2) $$\frac{dP(y)}{P(y)}=-\frac{2\left(H_{0}+D\right)}{H_{0}D}\mu dy$$

Now, applying the boundary condition $P(y=0)=P_{0}$, the pressure distribution within the left part of the sample is as follows:

(A3) $$P(y)=P_{0}\exp\left(-\frac{2\mu y\left(H_{0}+D\right)}{H_{0}D}\right)$$

Note that we considered that the pressure applied by the punch is penetrating into the metal as a hydrostatic stress, which can be justified by the fully constrained nature of this extrusion process.

The same above detailed calculation procedure leads to the following formula for the pressure distribution on the right side of the sample:

(A4) $$P_{BP}(y)=P_{BP}\exp\left(\frac{2\mu y\left(H_{1}+D\right)}{H_{1}D}\right)\;$$

Here, *y* is measured from the right-hand end of the specimen. Note that the change in sign of the exponent in Equation (A4) compared with Equation (A3) is because of the fact that the friction force is parallel to the force of the applied back-pressure on the right-hand side.

As can be seen from Equations (A3) and (A4), the pressure decreases from the position of the pressing punch and increases from the back-pressure punch within the sample. The two pressures then have to give the same value at the position where the left and right parts of the sample meet; where the fin is formed. This physical condition permits to extract the following formula for the friction:

(A5) $$\mu =\frac{D}{2}\ln\left(\frac{P_{0}}{P_{BP}}\right){\left[\frac{L_{1}\left(H_{1}+D\right)}{H_{1}}+\frac{L_{00}\left(H_{0}+D\right)}{H_{0}}\right]}^{-1}$$

Here, $L_{00}$ and $L_{1}$ are the lengths of the left and right parts of the sample, respectively. The value of the pressure at the position of the fin can be obtained from Equation (A3), for the y value of $y=L_{00}$:

(A6) $$P_{fin}=P_{0}\exp\left(-\frac{2\mu L_{00}\left(H_{0}+D\right)}{H_{0}D}\right)$$

By substituting the formula for friction, we get the following:

(A7) $$P_{fin}=P_{0}\exp\left(-\frac{2L_{00}\left(H_{0}+D\right)}{H_{0}}\ln\left(\frac{P_{0}}{P_{BP}}\right){\left[\frac{L_{1}\left(H_{1}+D\right)}{H_{1}}+\frac{L_{00}\left(H_{0}+D\right)}{H_{0}}\right]}^{-1}\right)$$

The friction power generated on the left and right sides of the specimen can be readily derived with the help of the equations for the pressure. The increment of the power on a slice is as follows:

(A8) $$dW_{f0}=2\mu \left(H_{0}+D\right)P(y)U_{0}=2\mu \left(H_{0}+D\right)P_{0}\exp\left(-\frac{2\mu y\left(H_{0}+D\right)}{H_{0}D}\right)U_{0}$$

By integrating, we obtain the friction powers for the left and right parts of the sample:

(A9) $$W_{f0}=U_{0}H_{0}DP_{0}\left[1-\exp\left(\frac{-2\mu L_{00}\left(H_{0}+D\right)}{H_{0}D}\right)\right]\;$$

(A10) $$W_{fBP}=U_{1}H_{1}DP_{BP}\left[\exp\left(\frac{2\mu L_{1}\left(H_{1}+D\right)}{H_{1}D}-1\right)\right]\;$$

## Appendix B

### Minimization of the Total Power for Obtaining the x Value

We look for the minimum of the function:

(A11) $$f\left(x\right)=\frac{2}{\sqrt{3}}\left(1-x\right)\left|r-x\right|+\frac{x}{\sqrt{3}}\left(\frac{x}{\bar{h}}+\frac{\bar{h}}{x}\right)+{\bar{P}}_{BP}\left(1-x\right)+\mu_{DMZ}{\bar{P}}_{fin}\frac{x^{2}}{\bar{h}}+{\bar{P}}_{BP}(1-x)\left[\exp\left(\frac{2\mu L_{1}\left(H_{1}+D\right)}{H_{1}D}\right)-1\right]$$

Because of the absolute value expression in this function, we examine two cases: x<r and x≥r.

1. x<r

(A12) $$f\left(x\right)=\frac{2}{\sqrt{3}}\left(1-x\right)(r-x)+\frac{x}{\sqrt{3}}\left(\frac{x}{\bar{h}}+\frac{\bar{h}}{x}\right)+{\bar{P}}_{BP}\left(1-x\right)+\mu_{DMZ}{\bar{P}}_{fin}\frac{x^{2}}{\bar{h}}+{\bar{P}}_{BP}(1-x)\left[\exp\left(\frac{2\mu L_{1}\left(H_{1}+D\right)}{H_{1}D}\right)-1\right]\;$$

The minimum is determined by solving the equation $\frac{df(x)}{dx}=0$:

(A13) $$\begin{array}{l} \frac{df(x)}{dx}=-\frac{2}{\sqrt{3}}\left(r-x\right)-\frac{2}{\sqrt{3}}\left(1-x\right)+\frac{1}{\sqrt{3}}\left(\frac{x}{\bar{h}}+\frac{\bar{h}}{x}\right)+\frac{x}{\sqrt{3}}\left(\frac{1}{\bar{h}}-\frac{\bar{h}}{x^{2}}\right)-{\bar{P}}_{BP}+\mu_{DMZ}{\bar{P}}_{fin}\frac{2x}{\bar{h}}-{\bar{P}}_{BP}\left[\exp\left(\frac{2\mu L_{1}\left(H_{1}+D\right)}{H_{1}D}\right)-1\right]\\ =-\frac{2}{\sqrt{3}}\left(r-x\right)-\frac{2}{\sqrt{3}}\left(1-x\right)+\frac{2}{\sqrt{3}}\frac{x}{\bar{h}}+\mu_{DMZ}{\bar{P}}_{fin}\frac{2x}{\bar{h}}-\exp\left(\frac{2\mu L_{1}\left(H_{1}+D\right)}{H_{1}D}\right){\bar{P}}_{BP}=0 \end{array}\;$$

This results in the following:

(A14) $$x=\bar{h}\left\{\left(1+r\right)+\frac{\sqrt{3}}{2}\exp\left(\frac{2\mu L_{1}\left(H_{1}+D\right)}{H_{1}D}\right){\bar{P}}_{BP}\right\}{\left(1+2\bar{h}+\sqrt{3}\mu_{DMZ}{\bar{P}}_{fin}\right)}^{-1}\;$$

Because we examine the case x<r, this expression is valid when

(A15) $$\bar{h}\left[\left(1+r\right)+\frac{\sqrt{3}}{2}\exp\left(\frac{2\mu L_{1}\left(H_{1}+D\right)}{H_{1}D}\right){\bar{P}}_{BP}\right]{\left(1+2\bar{h}+\sqrt{3}\mu_{DMZ}{\bar{P}}_{fin}\right)}^{-1}<r\;$$

This implies that ${\bar{P}}_{BP}<{\bar{P}}_{BP1}$, where

(A16) $${\bar{P}}_{BP1}=\left[\frac{r}{\bar{h}}(1+2\bar{h}+\sqrt{3}\mu_{DMZ}{\bar{P}}_{fin})-(1+r)\right]{\left[\frac{\sqrt{3}}{2}\exp\left(\frac{2\mu L_{1}\left(H_{1}+D\right)}{H_{1}D}\right)\right]}^{-1}\;$$

If ${\bar{P}}_{BP}={\bar{P}}_{BP1}$, then x=r.

If ${\bar{P}}_{BP}>{\bar{P}}_{BP1}$, Equation (A13) does not have a solution satisfying condition x<r.

This means that the minimum of the function (A12) is attained at the point x=r.

2. x≥r

(A17) $$f\left(x\right)=-\frac{2}{\sqrt{3}}\left(1-x\right)\left(r-x\right)+\frac{x}{\sqrt{3}}\left(\frac{x}{\bar{h}}+\frac{\bar{h}}{x}\right)+{\bar{P}}_{BP}\left(1-x\right)+\mu_{DMZ}{\bar{P}}_{fin}\frac{x^{2}}{\bar{h}}+{\bar{P}}_{BP}(1-x)\left[\exp\left(\frac{2\mu L_{1}\left(H_{1}+D\right)}{H_{1}D}\right)-1\right]\;$$

The minimum is determined by solving equation $\frac{df(x)}{dx}=0$.

(A18) $$\begin{array}{l} \frac{df(x)}{dx}=\frac{2}{\sqrt{3}}\left(r-x\right)+\frac{2}{\sqrt{3}}\left(1-x\right)+\frac{1}{\sqrt{3}}\left(\frac{x}{\bar{h}}+\frac{\bar{h}}{x}\right)+\frac{x}{\sqrt{3}}\left(\frac{1}{\bar{h}}-\frac{\bar{h}}{x^{2}}\right)-{\bar{P}}_{BP}+\mu_{DMZ}{\bar{P}}_{fin}\frac{2x}{\bar{h}}-{\bar{P}}_{BP}\left[\exp\left(\frac{2\mu L_{1}\left(H_{1}+D\right)}{H_{1}D}\right)-1\right]\\ =\frac{2}{\sqrt{3}}\left(r-x\right)+\frac{2}{\sqrt{3}}\left(1-x\right)+\frac{2}{\sqrt{3}}\frac{x}{\bar{h}}-{\bar{P}}_{BP}+\mu_{DMZ}{\bar{P}}_{fin}\frac{2x}{\bar{h}}-{\bar{P}}_{BP}\exp\left(\frac{2\mu L_{1}\left(H_{1}+D\right)}{H_{1}D}\right)=0 \end{array}\;$$

This results in the following:

(A19) $$x=\bar{h}\left\{-\left(1+r\right)+\frac{\sqrt{3}}{2}\exp\left(\frac{2\mu L_{1}\left(H_{1}+D\right)}{H_{1}D}\right){\bar{P}}_{BP}\right\}{\left(1-2\bar{h}+\sqrt{3}\mu_{DMZ}{\bar{P}}_{fin}\right)}^{-1}\;$$

Because we examine the case x≥r, this expression is valid when

(A20) $$\bar{h}\left\{-\left(1+r\right)+\frac{\sqrt{3}}{2}\exp\left(\frac{2\mu L_{1}\left(H_{1}+D\right)}{H_{1}D}\right){\bar{P}}_{BP}\right\}{\left(1-2\bar{h}+\sqrt{3}\mu_{DMZ}{\bar{P}}_{fin}\right)}^{-1}\geq r\;$$

This indicates that ${\bar{P}}_{BP}>{\bar{P}}_{BP2}$, where

(A21) $${\bar{P}}_{BP2}=\left[\frac{r}{\bar{h}}(1-2\bar{h}+\sqrt{3}\mu_{DMZ}{\bar{P}}_{fin})+(1+r)\right]{\left[\frac{\sqrt{3}}{2}\exp\left(\frac{2\mu L_{1}\left(H_{1}+D\right)}{H_{1}D}\right)\right]}^{-1}\;$$

If ${\bar{P}}_{BP}={\bar{P}}_{BP2}$, then x=r.

If ${\bar{P}}_{BP}<{\bar{P}}_{BP2}$, Equation (A8) does not have a solution satisfying condition x>r. This means that the minimum of the function (2) is attained at the point x=r.

Combining cases 1 and 2, we obtain the relations (41)–(45) above.
